# 3-Ethyl-4-[3-(1*H*-imidazol-1-yl)prop­yl]-5-phenyl-4*H*-1,2,4-triazole dihydrate

**DOI:** 10.1107/S160053681003936X

**Published:** 2010-10-09

**Authors:** Anuradha Gurumoorthy, Vasuki Gopalsamy, Dilek Ünlüer, Esra Düğdü, Babu Varghesee

**Affiliations:** a Department of Physics, Saveetha School of Engineering, Saveetha University, Chennai-5, India; b Department of Physics, Kunthavai Naachiar Government Arts College for Women (Autonomous), Thanjavur 7, India; c Department of Chemistry, Faculty of Arts and Sciences, Karadeniz Teknik University, Trabzon 61080, Turkey; d Sophisticated Analytical Instrumentation Facilities, Indian Institute of Technology, Madras, Chennai 36, India

## Abstract

In the title compound, C_16_H_19_N_5_·2H_2_O, the triazole ring makes dihedral angles of 70.61 (6) and 41.89 (8)°, respectively, with the imidazole and benzene rings. The water mol­ecules are involved in inter­molecular O—H⋯N and O—H⋯O hydrogen bonds, which stabilize the crystal packing.

## Related literature

For a related structure, see: Rizzoli *et al.* (2009 ▶); Kalkan *et al.* (2007 ▶). For bond lengths and angles in triazole rings, see: Thenmozhi *et al.* (2010 ▶); Rizzoli *et al.* (2009 ▶); Dolzhenko *et al.* (2010 ▶); Ocak Ískeleli *et al.* (2005 ▶); Ünver *et al.* (2010 ▶). For the biological activity of triazole Schiff bases, see: Thenmozhi *et al.* (2010 ▶) and of 1,2,4-triazole derivatives, see: Ünver *et al.* (2010 ▶). For the search for and synthesis of new anti­biotics, see: Köysal *et al.* (2006 ▶). For the synthesis, see: Ünver *et al.* (2009 ▶). 
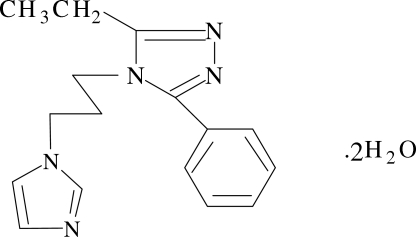


## Experimental

### 

#### Crystal data

C_16_H_19_N_5_·2H_2_O
*M* *_r_* = 317.39
Monoclinic, 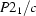

*a* = 11.0787 (16) Å
*b* = 9.8428 (8) Å
*c* = 16.3289 (18) Å
β = 105.602 (9)°
*V* = 1715.0 (3) Å^3^
*Z* = 4
Cu *K*α radiation
μ = 0.68 mm^−1^
*T* = 293 K
0.30 × 0.20 × 0.20 mm

#### Data collection

Enraf–Nonius CAD-4 diffractometer
Absorption correction: ψ scan North *et al.* (1968 ▶) *T*_min_ = 0.821, *T*_max_ = 0.876
3030 measured reflections
2868 independent reflections
2166 reflections with *I* > 2σ(*I*)
*R*_int_ = 0.029
2 standard reflections every 60 min  intensity decay: none

#### Refinement

*R*[*F* ^2^ > 2σ(*F* ^2^)] = 0.045
*wR*(*F* ^2^) = 0.123
*S* = 1.08
2868 reflections
226 parameters
6 restraints
H atoms treated by a mixture of independent and constrained refinement
Δρ_max_ = 0.19 e Å^−3^
Δρ_min_ = −0.18 e Å^−3^

### 

Data collection: *CAD-4 EXPRESS* (Enraf–Nonius, 1994 ▶); cell refinement: *CAD-4 EXPRESS*; data reduction: *MolEN* (Fair, 1990 ▶); program(s) used to solve structure: *SHELXS97* (Sheldrick, 2008 ▶); program(s) used to refine structure: *SHELXL97* (Sheldrick, 2008 ▶); molecular graphics: *ZORTEP* (Zsolnai, 1997 ▶); software used to prepare material for publication: *SHELXL97*.

## Supplementary Material

Crystal structure: contains datablocks I, global. DOI: 10.1107/S160053681003936X/jh2213sup1.cif

Structure factors: contains datablocks I. DOI: 10.1107/S160053681003936X/jh2213Isup2.hkl

Additional supplementary materials:  crystallographic information; 3D view; checkCIF report

## Figures and Tables

**Table:** 

C7—N1	1.308 (2)
C7—N3	1.373 (2)
C8—N2	1.312 (2)
C8—N3	1.363 (2)
N1—N2	1.387 (2)

**Table:** 

C8—N3—C7	105.09 (14)
C8—N3—C11	126.52 (14)
C7—N3—C11	128.05 (14)

**Table 2:** Hydrogen-bond geometry (Å, °)

*D*—H⋯*A*	*D*—H	H⋯*A*	*D*⋯*A*	*D*—H⋯*A*
O1—H1*A*⋯O2	0.91 (1)	1.98 (1)	2.882 (3)	172 (3)
O1—H1*B*⋯N2^i^	0.90 (1)	2.02 (1)	2.913 (2)	170 (3)
O2—H2*A*⋯N5^ii^	0.91 (1)	1.95 (1)	2.859 (3)	176 (2)
O2—H2*B*⋯O1^iii^	0.91 (1)	2.08 (2)	2.949 (3)	160 (3)

Symmetry codes: (i) 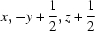; (ii) 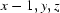; (iii) 
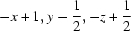
.

**Fig. 1.**
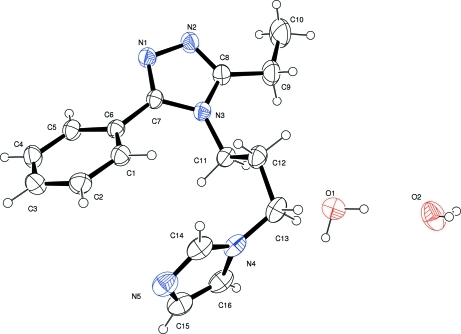
The molecular structure of the title compound. Displacement ellipsoids are drawn at the 50% probability level.

**Fig. 2.**
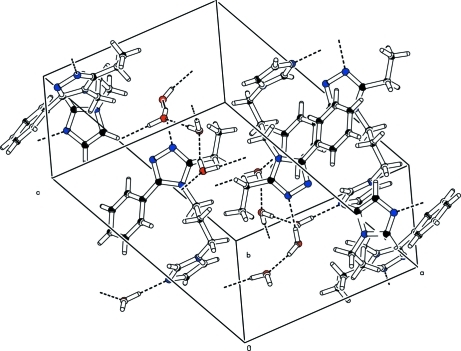
Crystal packing of the title compound viewed along the a axiS. Intermolecular O—H···O and O—H···N hydrogen bonds are shown as dashed lines.

**Fig. 3.**
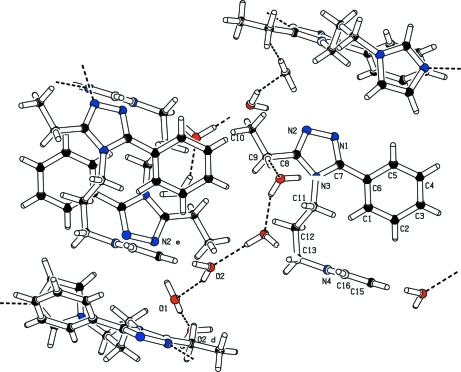
Crystal Packing of the title compound with hydrogen bonds.

## supplementary crystallographic information

### Comment

The biological importance of imidazoles and triazoles has stimulated much work on these heterocycles. 1,2,4-Triazole is a basic aromatic ring and possesses good coordination ability due to the presence of nitrogen atoms (Thenmozhi *et al.*,2010). 1,2,4-Triazole compounds posses important pharmacology activities such as antifungal and antiviral activities. Examples of such compounds bearing the 1,2,4-Triazole residues are fluconazole, the powerful azole antifungal agent as well as the potent antiviral N-nucleoside ribavirin. Furthermore various 1,2,4-Triazole derivatives have been reported as fungicidal, insecticidal, antimicrobial as well as anticonvulsants, antidepressants and plant growth regulator anticoagulants (Ünver *et al.*, 2010). 1,2,4-Triazole derivatives are also used to build polymetallic complexes. Compounds derived from triazole possess antimicrobial, analgesic, anti-inflammatory, local anesthetic, antineoplastic and antimalarial properties. Some triazole Schiff bases also exhibit antiproliferative and anticancer activities (Thenmozhi *et al.*, 2010). 1,2,4-Triazole moieties interact strongly with heme iron and aromatic substituents on the triazoles are very effective for interacting with the active site of aromatase. Furthermore, it was reported that compounds having triazole moieties such as Vorozole, Anastrozole and Letrozole appear to be very effective aromatese inhibitors very useful for preventing breast cancer (Ünver *et al.*, 2010). Some of the azole derivatives used as common antibiotics, such as amphotericin B, exhibit toxic effects on humans along with antimicrobial effects. Although different antimicrobial agents are used in the treatment of microbial infections, an increasing resistance to these drugs is observed. Therefore, the search for and synthesis of new antibiotics different from commonly used ones is of current importance (Köysal *et al.*, 2006). In a search for new triazole compounds with better biological activity, the title compound (I), was synthesized. We report here the crystal structure of the title compound, (I) (Fig.1), a new 1,2,4-triazole derivative. The compound (I) crystallizes as a dihydrate, the bond lengths and angles (Table 1) are generally normal in the triazole ring (Thenmozhi *et al.*, 2010; Rizzoli *et al.*, 2009; Dolzhenko *et al.*, 2010; Ocak Ískeleli *et al.*, 2005; Ünver *et al.*, 2010). Atom N3 has a trigonal configuration, the sum of the three bond angles around them being 360° (Kalkan *et al.*, 2007). The dihedral angles between the planes A(N1/N2/C7/N3/C8), B(N4/C14/N5/C15/C16) and C(C1/C2/C3/C4/C5/C6) are A/B = 70.61 (6)°, A/C = 41.89 (8)° and B/C = 68.16 (7)°. The triazole ring is essentially planar with r.m.s deviation of 0.0046Å (Dolzhenko *et al.*, 2010). The C—N bond lengths in the triazole ring of all molecules lie in the range of 1.260 (3)–1.349 (4) Å. These are longer than a typical double C═ N bond [1.269 (2) Å], but shorter than a C—N single bond [1.443 (4) Å], (Thenmozhi *et al.*, 2010) indicating the possibility of electron delocalization. The N3–C11–C12–C13 torsion angle of 172.00 (15)° indicates that the triazole ring and the imidazole moiety has an E-configuration across the C11–C12 bond. This configuration is stabilized by an intramolecular C11–H11···N4 hydrogen bond with H11···N4 distance of 2.54 Å. The uncoordinated water molecules are involved in intermolecular O–H···O and O–H···N hydrogen bonds (Table 2), which stabilize the crystal packing.

### Experimental

The compound was synthesized by published method (Ünver *et al.*, 2009).

### Refinement

Water H atoms were located in a difference Fourier map and isotropically refined with O—H distance restraints of 0.90 (1) Å. All the other H atoms were positioned geometrically and treated as riding on their parent atoms, with C—H = 0.93Å (aromatic) and 0.97Å (methylene), N—H = 0.86 Å, and refined using a riding model with *U*_iso_(H) = 1.2*U*_eq_ or 1.5*U*_eq_(parent atom). In the absence of significant anomalous scattering effects, Friedel pairs were merged.

### Crystal data

**Table:**

C_16_H_19_N_5_·2H_2_O	*F*(000) = 680
*M**_r_* = 317.39	*D*_x_ = 1.229 Mg m^−^^3^
Monoclinic, *P*2_1_/*c*	Cu *K*α radiation, λ = 1.54180 Å
*a* = 11.0787 (16) Å	Cell parameters from 25 reflections
*b* = 9.8428 (8) Å	θ = 20–30°
*c* = 16.3289 (18) Å	µ = 0.68 mm^−^^1^
β = 105.602 (9)°	*T* = 293 K
*V* = 1715.0 (3) Å^3^	Block, colourless
*Z* = 4	0.30 × 0.20 × 0.20 mm

### Data collection

**Table:**

Enraf–Nonius CAD-4 Diffractometer	2166 reflections with *I* > 2σ(*I*)
Radiation source: fine-focus sealed tube	*R*_int_ = 0.029
graphite	θ_max_ = 64.9°, θ_min_ = 4.1°
ω–2τ scan	*h* = 0→13
Absorption correction: ψ scan North *et al.* (1968)	*k* = 0→11
*T*_min_ = 0.821, *T*_max_ = 0.876	*l* = −19→18
3030 measured reflections	2 standard reflections every 60 min
2868 independent reflections	intensity decay: none

### Refinement

**Table:**

Refinement on *F*^2^	Secondary atom site location: difference Fourier map
Least-squares matrix: full	Hydrogen site location: inferred from neighbouring sites
*R*[*F*^2^ > 2σ(*F*^2^)] = 0.045	H atoms treated by a mixture of independent and constrained refinement
*wR*(*F*^2^) = 0.123	*w* = 1/[σ^2^(*F*_o_^2^) + (0.0676*P*)^2^ + 0.2107*P*] where *P* = (*F*_o_^2^ + 2*F*_c_^2^)/3
*S* = 1.08	(Δ/σ)_max_ < 0.001
2868 reflections	Δρ_max_ = 0.19 e Å^−^^3^
226 parameters	Δρ_min_ = −0.17 e Å^−^^3^
6 restraints	Extinction correction: *SHELXL*, Fc^*^=kFc[1+0.001xFc^2^λ^3^/sin(2θ)]^-1/4^
Primary atom site location: structure-invariant direct methods	Extinction coefficient: 0.0246 (12)

### Special details

**Table:**

Experimental. Number of psi-scan sets used was 5 Theta correction was applied. Averaged transmission function was used. No Fourier smoothing was applied.
Geometry. All e.s.d.'s (except the e.s.d. in the dihedral angle between two l.s. planes) are estimated using the full covariance matrix. The cell e.s.d.'s are taken into account individually in the estimation of e.s.d.'s in distances, angles and torsion angles; correlations between e.s.d.'s in cell parameters are only used when they are defined by crystal symmetry. An approximate (isotropic) treatment of cell e.s.d.'s is used for estimating e.s.d.'s involving l.s. planes.
Refinement. Refinement of *F*^2^ against ALL reflections. The weighted *R*-factor *wR* and goodness of fit *S* are based on *F*^2^, conventional *R*-factors *R* are based on *F*, with *F* set to zero for negative *F*^2^. The threshold expression of *F*^2^ > σ(*F*^2^) is used only for calculating *R*-factors(gt) *etc*. and is not relevant to the choice of reflections for refinement. *R*-factors based on *F*^2^ are statistically about twice as large as those based on *F*, and *R*- factors based on ALL data will be even larger.

### Fractional atomic coordinates and isotropic or equivalent isotropic displacement parameters (Å^2^)

**Table:**

	*x*	*y*	*z*	*U*_iso_*/*U*_eq_	
C1	1.10619 (16)	0.09615 (18)	0.08555 (11)	0.0519 (4)	
H1	1.0554	0.0218	0.0878	0.062*	
C2	1.23425 (18)	0.0845 (2)	0.11690 (12)	0.0609 (5)	
H2	1.2692	0.0026	0.1400	0.073*	
C3	1.31051 (18)	0.1935 (2)	0.11417 (12)	0.0655 (6)	
H3	1.3970	0.1855	0.1354	0.079*	
C4	1.25803 (18)	0.3152 (2)	0.07964 (12)	0.0623 (5)	
H4	1.3094	0.3893	0.0779	0.075*	
C5	1.13010 (17)	0.32727 (17)	0.04779 (11)	0.0509 (4)	
H5	1.0956	0.4092	0.0242	0.061*	
C6	1.05207 (15)	0.21742 (16)	0.05067 (10)	0.0429 (4)	
C7	0.91650 (15)	0.23322 (16)	0.01343 (10)	0.0430 (4)	
C8	0.71432 (16)	0.23090 (19)	−0.00821 (11)	0.0529 (5)	
C9	0.58650 (18)	0.2091 (3)	0.00312 (15)	0.0772 (6)	
H9A	0.5689	0.1124	0.0005	0.093*	
H9B	0.5856	0.2406	0.0592	0.093*	
C10	0.4853 (2)	0.2794 (3)	−0.06124 (19)	0.1100 (10)	
H10A	0.5027	0.3750	−0.0600	0.165*	
H10B	0.4066	0.2646	−0.0486	0.165*	
H10C	0.4813	0.2441	−0.1167	0.165*	
C11	0.83527 (17)	0.11825 (17)	0.12819 (10)	0.0496 (4)	
H11A	0.7754	0.1576	0.1551	0.060*	
H11B	0.9185	0.1359	0.1648	0.060*	
C12	0.81484 (18)	−0.03413 (18)	0.12166 (11)	0.0571 (5)	
H12A	0.8667	−0.0732	0.0884	0.069*	
H12B	0.7279	−0.0529	0.0925	0.069*	
C13	0.8473 (2)	−0.0997 (2)	0.20926 (13)	0.0658 (5)	
H13A	0.7918	−0.0643	0.2411	0.079*	
H13B	0.8335	−0.1969	0.2029	0.079*	
C14	1.0814 (2)	−0.1225 (2)	0.23923 (13)	0.0618 (5)	
H14	1.0804	−0.1814	0.1945	0.074*	
C15	1.1436 (2)	0.0064 (2)	0.34639 (14)	0.0700 (6)	
H15	1.1959	0.0543	0.3912	0.084*	
C16	1.0180 (2)	0.0092 (2)	0.32597 (12)	0.0655 (5)	
H16	0.9685	0.0584	0.3532	0.079*	
N1	0.86819 (13)	0.29858 (15)	−0.05759 (9)	0.0524 (4)	
N2	0.73910 (14)	0.29737 (16)	−0.07142 (10)	0.0572 (4)	
N3	0.82282 (12)	0.18678 (13)	0.04670 (8)	0.0455 (4)	
N4	0.97689 (15)	−0.07477 (15)	0.25699 (9)	0.0564 (4)	
N5	1.18406 (17)	−0.07686 (18)	0.29187 (11)	0.0692 (5)	
O1	0.60569 (16)	0.10016 (18)	0.26136 (10)	0.0807 (5)	
O2	0.43707 (15)	−0.11055 (19)	0.28415 (13)	0.0920 (6)	
H1A	0.554 (2)	0.030 (2)	0.2642 (16)	0.127 (11)*	
H1B	0.650 (2)	0.121 (3)	0.3147 (9)	0.134 (11)*	
H2A	0.3579 (13)	−0.096 (2)	0.2882 (17)	0.105 (9)*	
H2B	0.444 (2)	−0.2016 (12)	0.277 (2)	0.154 (14)*	

### Atomic displacement parameters (Å^2^)

**Table:**

	*U*^11^	*U*^22^	*U*^33^	*U*^12^	*U*^13^	*U*^23^
C1	0.0520 (10)	0.0489 (10)	0.0551 (10)	−0.0035 (8)	0.0146 (8)	0.0050 (8)
C2	0.0562 (11)	0.0673 (12)	0.0575 (11)	0.0090 (9)	0.0124 (8)	0.0122 (9)
C3	0.0467 (11)	0.0881 (15)	0.0583 (11)	−0.0024 (10)	0.0086 (8)	−0.0020 (10)
C4	0.0537 (11)	0.0680 (12)	0.0668 (12)	−0.0186 (9)	0.0190 (9)	−0.0075 (10)
C5	0.0543 (11)	0.0451 (9)	0.0560 (10)	−0.0047 (8)	0.0193 (8)	−0.0015 (8)
C6	0.0464 (10)	0.0417 (8)	0.0423 (8)	−0.0029 (7)	0.0152 (7)	−0.0023 (7)
C7	0.0455 (9)	0.0376 (8)	0.0483 (9)	−0.0039 (7)	0.0169 (7)	−0.0005 (7)
C8	0.0472 (10)	0.0528 (10)	0.0598 (10)	−0.0034 (8)	0.0163 (8)	0.0020 (8)
C9	0.0505 (12)	0.0929 (16)	0.0929 (15)	−0.0052 (11)	0.0271 (11)	0.0124 (13)
C10	0.0514 (14)	0.151 (3)	0.131 (2)	0.0045 (15)	0.0297 (14)	0.031 (2)
C11	0.0578 (10)	0.0464 (9)	0.0489 (9)	−0.0042 (8)	0.0218 (8)	−0.0010 (7)
C12	0.0665 (12)	0.0479 (10)	0.0615 (11)	−0.0122 (8)	0.0252 (9)	−0.0007 (8)
C13	0.0780 (14)	0.0530 (11)	0.0757 (13)	−0.0041 (10)	0.0366 (11)	0.0124 (10)
C14	0.0839 (15)	0.0492 (10)	0.0594 (11)	0.0147 (10)	0.0314 (11)	0.0078 (9)
C15	0.0886 (16)	0.0605 (12)	0.0631 (12)	0.0040 (11)	0.0240 (11)	0.0049 (10)
C16	0.0951 (16)	0.0532 (11)	0.0590 (11)	0.0097 (10)	0.0394 (11)	0.0041 (9)
N1	0.0461 (8)	0.0548 (9)	0.0562 (8)	−0.0011 (7)	0.0137 (6)	0.0098 (7)
N2	0.0439 (9)	0.0624 (9)	0.0638 (9)	−0.0005 (7)	0.0121 (7)	0.0096 (8)
N3	0.0477 (8)	0.0424 (7)	0.0485 (7)	−0.0045 (6)	0.0163 (6)	0.0002 (6)
N4	0.0741 (11)	0.0474 (8)	0.0559 (8)	0.0066 (7)	0.0314 (8)	0.0092 (7)
N5	0.0786 (12)	0.0630 (11)	0.0704 (10)	0.0143 (9)	0.0278 (9)	0.0130 (9)
O1	0.0767 (11)	0.0899 (12)	0.0706 (10)	−0.0057 (9)	0.0114 (8)	0.0019 (8)
O2	0.0619 (10)	0.0805 (12)	0.1268 (15)	0.0036 (8)	0.0135 (9)	−0.0038 (10)

### Geometric parameters (Å, °)

**Table:**

C1—C2	1.377 (2)	C11—N3	1.465 (2)
C1—C6	1.387 (2)	C11—C12	1.516 (2)
C1—H1	0.9300	C11—H11A	0.9700
C2—C3	1.373 (3)	C11—H11B	0.9700
C2—H2	0.9300	C12—C13	1.522 (3)
C3—C4	1.384 (3)	C12—H12A	0.9700
C3—H3	0.9300	C12—H12B	0.9700
C4—C5	1.377 (3)	C13—N4	1.459 (2)
C4—H4	0.9300	C13—H13A	0.9700
C5—C6	1.393 (2)	C13—H13B	0.9700
C5—H5	0.9300	C14—N5	1.307 (3)
C6—C7	1.469 (2)	C14—N4	1.352 (2)
C7—N1	1.308 (2)	C14—H14	0.9300
C7—N3	1.373 (2)	C15—C16	1.341 (3)
C8—N2	1.312 (2)	C15—N5	1.371 (3)
C8—N3	1.363 (2)	C15—H15	0.9300
C8—C9	1.492 (3)	C16—N4	1.372 (2)
C9—C10	1.485 (3)	C16—H16	0.9300
C9—H9A	0.9700	N1—N2	1.387 (2)
C9—H9B	0.9700	O1—H1A	0.906 (10)
C10—H10A	0.9600	O1—H1B	0.902 (10)
C10—H10B	0.9600	O2—H2A	0.909 (9)
C10—H10C	0.9600	O2—H2B	0.910 (10)
			
C2—C1—C6	120.80 (16)	N3—C11—H11A	108.6
C2—C1—H1	119.6	C12—C11—H11A	108.6
C6—C1—H1	119.6	N3—C11—H11B	108.6
C3—C2—C1	120.29 (18)	C12—C11—H11B	108.6
C3—C2—H2	119.9	H11A—C11—H11B	107.6
C1—C2—H2	119.9	C11—C12—C13	111.13 (15)
C2—C3—C4	119.64 (18)	C11—C12—H12A	109.4
C2—C3—H3	120.2	C13—C12—H12A	109.4
C4—C3—H3	120.2	C11—C12—H12B	109.4
C5—C4—C3	120.35 (18)	C13—C12—H12B	109.4
C5—C4—H4	119.8	H12A—C12—H12B	108.0
C3—C4—H4	119.8	N4—C13—C12	112.37 (15)
C4—C5—C6	120.35 (17)	N4—C13—H13A	109.1
C4—C5—H5	119.8	C12—C13—H13A	109.1
C6—C5—H5	119.8	N4—C13—H13B	109.1
C1—C6—C5	118.56 (16)	C12—C13—H13B	109.1
C1—C6—C7	122.80 (14)	H13A—C13—H13B	107.9
C5—C6—C7	118.59 (15)	N5—C14—N4	112.54 (18)
N1—C7—N3	110.00 (14)	N5—C14—H14	123.7
N1—C7—C6	123.22 (14)	N4—C14—H14	123.7
N3—C7—C6	126.76 (14)	C16—C15—N5	110.43 (19)
N2—C8—N3	110.02 (15)	C16—C15—H15	124.8
N2—C8—C9	125.15 (17)	N5—C15—H15	124.8
N3—C8—C9	124.82 (17)	C15—C16—N4	106.56 (17)
C10—C9—C8	113.94 (19)	C15—C16—H16	126.7
C10—C9—H9A	108.8	N4—C16—H16	126.7
C8—C9—H9A	108.8	C7—N1—N2	107.35 (13)
C10—C9—H9B	108.8	C8—N2—N1	107.52 (13)
C8—C9—H9B	108.8	C8—N3—C7	105.09 (14)
H9A—C9—H9B	107.7	C8—N3—C11	126.52 (14)
C9—C10—H10A	109.5	C7—N3—C11	128.05 (14)
C9—C10—H10B	109.5	C14—N4—C16	105.73 (17)
H10A—C10—H10B	109.5	C14—N4—C13	127.14 (17)
C9—C10—H10C	109.5	C16—N4—C13	127.00 (17)
H10A—C10—H10C	109.5	C14—N5—C15	104.73 (18)
H10B—C10—H10C	109.5	H1A—O1—H1B	108.4 (14)
N3—C11—C12	114.56 (14)	H2A—O2—H2B	106.5 (14)
			
C6—C1—C2—C3	0.1 (3)	C6—C7—N3—C8	177.38 (15)
C1—C2—C3—C4	−0.1 (3)	N1—C7—N3—C11	−174.85 (15)
C2—C3—C4—C5	−0.3 (3)	C6—C7—N3—C11	3.7 (3)
C3—C4—C5—C6	0.6 (3)	C12—C11—N3—C8	84.7 (2)
C2—C1—C6—C5	0.2 (3)	C12—C11—N3—C7	−102.89 (19)
C2—C1—C6—C7	177.55 (16)	N5—C14—N4—C16	0.8 (2)
C4—C5—C6—C1	−0.5 (2)	N5—C14—N4—C13	176.98 (15)
C4—C5—C6—C7	−177.98 (15)	C15—C16—N4—C14	−0.6 (2)
C1—C6—C7—N1	−137.55 (18)	C15—C16—N4—C13	−176.81 (16)
C5—C6—C7—N1	39.8 (2)	C12—C13—N4—C14	−67.3 (2)
C1—C6—C7—N3	44.1 (2)	C12—C13—N4—C16	108.1 (2)
C5—C6—C7—N3	−138.58 (17)	N4—C14—N5—C15	−0.6 (2)
N2—C8—C9—C10	−4.5 (3)	C16—C15—N5—C14	0.2 (2)
N3—C8—C9—C10	174.1 (2)	C7—N3—C11—C12	−102.89 (19)
N3—C11—C12—C13	172.00 (15)	C6—C7—N3—C11	3.7 (3)
C11—C12—C13—N4	−58.7 (2)	C6—C7—N3—C8	177.38 (15)
N5—C15—C16—N4	0.3 (2)	N1—C7—N3—C11	−174.85 (15)
N3—C7—N1—N2	0.85 (18)	N2—C8—N3—C11	174.88 (15)
C6—C7—N1—N2	−177.79 (14)	C9—C8—N3—C11	−3.9 (3)
N3—C8—N2—N1	−0.6 (2)	C5—C6—C7—N1	39.8 (2)
C9—C8—N2—N1	178.21 (19)	N1—C7—C6—C1	−137.55 (18)
C7—N1—N2—C8	−0.15 (19)	N3—C7—C6—C1	44.1 (2)
N2—C8—N3—C7	1.09 (19)	N2—C8—C9—C10	−4.5 (3)
C9—C8—N3—C7	−177.73 (19)	C10—C9—C8—N3	174.1 (2)
N2—C8—N3—C11	174.88 (15)	N4—C13—C12—C11	−58.7 (2)
C9—C8—N3—C11	−3.9 (3)	N3—C11—C12—C13	172.00 (15)
N1—C7—N3—C8	−1.19 (18)		

### Hydrogen-bond geometry (Å, °)

**Table:**

*D*—H···*A*	*D*—H	H···*A*	*D*···*A*	*D*—H···*A*
O1—H1A···O2	0.91 (1)	1.98 (1)	2.882 (3)	172 (3)
O1—H1B···N2^i^	0.90 (1)	2.02 (1)	2.913 (2)	170 (3)
O2—H2A···N5^ii^	0.91 (1)	1.95 (1)	2.859 (3)	176 (2)
O2—H2B···O1^iii^	0.91 (1)	2.08 (2)	2.949 (3)	160 (3)

Symmetry codes: (i) *x*, −*y*+1/2, *z*+1/2; (ii) *x*−1, *y*, *z*; (iii) −*x*+1, *y*−1/2, −*z*+1/2.

## References

[bb1] Dolzhenko, A. V., Tan, G. K., Koh, L. L., Dolzhenko, A. V. & Chui, W. K. (2010). *Acta Cryst.* E**66**, o425. 10.1107/S1600536810002369 PMC2979925 21579840

[bb2] Enraf–Nonius (1994). *CAD-4 EXPRESS* Enraf–Nonius, Delft, The Netherlands.

[bb3] Fair, C. K. (1990). *MolEN* Enraf–Nonius, Delft, The Netherlands.

[bb4] Kalkan, H., Ustabaş, R., Sancak, K., Ünver, Y. & Vázquez-López, E. M. (2007). *Acta Cryst.* E**63**, o2449–o2451.

[bb5] Köysal, Y., Işık, Ş., Sancak, K. & Ünver, Y. (2006). *Acta Cryst.* E**62**, o3907–o3909.

[bb6] North, A. C. T., Phillips, D. C. & Mathews, F. S. (1968). *Acta Cryst.* A**24**, 351–359.

[bb7] Ocak Ískeleli, N., Işık, S., Sancak, K., Şaşmaz, S., Ünver, Y. & Er, M. (2005). *Acta Cryst.* C**61**, o363–o365. 10.1107/S0108270105008449 15930685

[bb8] Rizzoli, C., Marku, E. & Greci, L. (2009). *Acta Cryst.* E**65**, o663. 10.1107/S1600536809007120 PMC2969042 21582408

[bb9] Sheldrick, G. M. (2008). *Acta Cryst.* A**64**, 112–122. 10.1107/S0108767307043930 18156677

[bb10] Thenmozhi, M., Kavitha, T., Reddy, B. P., Vijayakumar, V. & Ponnuswamy, M. N. (2010). *Acta Cryst.* E**66**, o558. 10.1107/S1600536810003946 PMC2983682 21580327

[bb11] Ünver, Y., Köysal, Y., Tanak, H., Ünlüer, D. & Işık, Ş. (2010). *Acta Cryst.* E**66**, o1294. 10.1107/S160053681001603X PMC2979362 21579391

[bb12] Ünver, Y., Sancak, K., Tanak, H., Değirmencioğlu, I., Düğdü, E., Er, M. & Işık, Ş. (2009). *J. Mol. Struct.* **936**, 46–55.

[bb13] Zsolnai, L. (1997). *ZORTEP* University of Heidelberg, Germany.

